# Modeling the Mechanism of Action of a DGAT1 Inhibitor Using a Causal Reasoning Platform

**DOI:** 10.1371/journal.pone.0027009

**Published:** 2011-11-04

**Authors:** Ahmed E. Enayetallah, Daniel Ziemek, Michael T. Leininger, Ranjit Randhawa, Jianxin Yang, Tara B. Manion, Dawn E. Mather, William J. Zavadoski, Max Kuhn, Judith L. Treadway, Shelly Ann G. des Etages, E. Michael Gibbs, Nigel Greene, Claire M. Steppan

**Affiliations:** 1 Compound Safety Prediction Group, Pfizer Inc., Groton, Connecticut, United States of America; 2 Computational Sciences Center of Emphasis, Pfizer Inc., Cambridge, Massachusetts, United States of America; 3 Cardiovascular, Metabolic and Endocrine Diseases Research Unit, Pfizer Inc., Groton, Connecticut, United States of America; Pennington Biomedical Research Center, United States of America

## Abstract

Triglyceride accumulation is associated with obesity and type 2 diabetes. Genetic disruption of diacylglycerol acyltransferase 1 (DGAT1), which catalyzes the final reaction of triglyceride synthesis, confers dramatic resistance to high-fat diet induced obesity. Hence, DGAT1 is considered a potential therapeutic target for treating obesity and related metabolic disorders. However, the molecular events shaping the mechanism of action of DGAT1 pharmacological inhibition have not been fully explored yet. Here, we investigate the metabolic molecular mechanisms induced in response to pharmacological inhibition of DGAT1 using a recently developed computational systems biology approach, the Causal Reasoning Engine (CRE). The CRE algorithm utilizes microarray transcriptomic data and causal statements derived from the biomedical literature to infer upstream molecular events driving these transcriptional changes. The inferred upstream events (also called *hypotheses*) are aggregated into biological models using a set of analytical tools that allow for evaluation and integration of the hypotheses in context of their supporting evidence. In comparison to gene ontology enrichment analysis which pointed to high-level changes in metabolic processes, the CRE results provide detailed molecular hypotheses to explain the measured transcriptional changes. CRE analysis of gene expression changes in high fat habituated rats treated with a potent and selective DGAT1 inhibitor demonstrate that the majority of transcriptomic changes support a metabolic network indicative of reversal of high fat diet effects that includes a number of molecular hypotheses such as PPARG, HNF4A and SREBPs. Finally, the CRE-generated molecular hypotheses from DGAT1 inhibitor treated rats were found to capture the major molecular characteristics of DGAT1 deficient mice, supporting a phenotype of decreased lipid and increased insulin sensitivity.

## Introduction

Triacylglycerol is a highly efficient energy storage form critical for surviving periods of starvation and extended physical activity. Many industrialized societies maintain a diet rich in fat and carbohydrates, and a sedentary lifestyle leading to the excess storage of triglyceride in tissues. The resulting adiposity has been linked to an increased prevalence of multiple diseases such as diabetes and atherosclerosis [1]. Diacylglycerol Acyltransferase (DGAT) enzymes catalyze the final step in the synthesis of triacylglycerol from diacylglycerol (DAG) and fatty acyl-coA making them attractive targets for reducing triglyceride storage [2]. Two separate genes encode for the DGAT1 and DGAT2 enzymes [3]. DGAT1 knock out (-/-) mice are lean, resistant to diet induced obesity and have increased insulin sensitivity, while the DGAT2 (-/-) genotype is lethal [4], [5]. Interestingly, intestine selective overexpression of DGAT1 in the context of mice null for DGAT1 reverses the lean phenotype and hepatic steatosis suggestive that DGAT1 expression in intestine is a major contributor to metabolic phenotype [6]. Out of a broad panel of human tissues DGAT1 was most abundant in the small intestine [2] We have confirmed these findings in human, rat and mouse tissues with gene chip profiling and RT-qPCR (data not shown). Therefore we sought to investigate the molecular changes occurring in the small intestine with pharmacological inhibition of DGAT1.

Whole genome expression measurements provide snapshots of the abundance of thousands of transcripts and have the potential to paint a comprehensive picture of modulated biological processes in a given sample. While most problems relating to the statistically robust estimation of transcript levels changing between different samples have been successfully solved, the task of manually interpreting the usually hundreds of changing transcript levels is daunting. At the same time, the amount of biomedical knowledge is growing rapidly. The PubMed database comprises more than 20 million citations as of October 2010 [7]. Methods that harness this knowledge for the interpretation of gene expression data are promising candidates to make the biological interpretation process as routine in the future as the statistical analysis of the transcript level changes is today.

The most popular class of methods to analyze gene expression data using pre-defined categories of genes (e.g. pathways, biological processes) is called *gene-set enrichment analysis*. Ackermann & Strimmer give an excellent recent review of the many methods proposed [8]. Gene-set enrichment methods provide a good first overview of high-level processes changing between measured conditions, but oftentimes lack the ability to provide concrete molecular hypotheses as to the causal drivers of the processes as well as direct suggestions for experimental follow-up. In this article, we focus on the use of a novel causal reasoning algorithm to infer upstream molecular mechanisms that caused observed expression changes. Causal reasoning algorithms can be viewed as a form of gene set enrichment with two major enhancements. First, such methods provide predictions on causal drivers on a molecular level by using gene sets corresponding to the effects of defined causal perturbations. Second, they account for directionality of the gene expression changes and hence the directionality of the inferred upstream molecular causes can be computed as well. Similar causal reasoning-based approaches have been described in the work of Pollard et al [9]. Here, we rely on a novel algorithm, called the Causal Reasoning Engine introduced by Chindelevitch et al, 2010 [10].

To increase our understanding of a novel DGAT1 inhibitor, PF-04620110 and its mechanism of action we monitored gene expression changes in the jejunum of rats following an acute exposure to PF-04620110. The gene expression changes were used by the causal reasoning platform to infer the molecular events shaping the biological response.

## Materials and Methods

### Ethics Statement

All in vivo procedures were reviewed and approved by the Institutional Animal Care and Use Committee at Pfizer Inc. (AUP# 3573).

### Animal Experiments

Male Sprague Dawley rats (Charles River Laboratories) (average initial body weight of approximately 250 grams) were individually housed in hanging wire mesh cages and acclimated to a reverse light/dark cycle (lights off at 10:00 am/lights on at 10:00 pm) and high fat Western diet (Research Diets D12079B) for 14 days prior to experimentation. Rats (n = 8 per treatment group) were randomized to receive vehicle or compound PF-04620110 at 3 or 15 mg/kg. PF-04620110 is a highly potent and selective inhibitor of DGAT1 with an IC50 of 19 nM at human DGAT1 and IC50 of 64 nM at rat DGAT1 [11]. PF-04620110 also possesses greater than 100-fold selectivity against hDGAT2, hACAT1, hAWAT1/2, hMGAT2/3, mMGAT1 [11].

Food was removed from each cage 24 hours prior to administration of DGAT1 inhibitor. Vehicle or test compound was administered via standard oral gavage in a dose volume of 5 mL/kg and rats were allowed ad libidum access to food and water for six hours, during which time food intake was monitored. After six hours animals were sacrificed for blood and tissue collection. Tissue samples (jejunum, liver) were flash frozen in liquid nitrogen. Blood was collected via cardiac puncture and placed into vacutainer EDTA tubes containing Aprotinin and DPP-IV inhibitor for plasma isolation. Triglyceride concentration in plasma samples were determined on a Roche 912 clinical chemistry analyzer (Roche Diagnostics). Plasma samples treated with Aprotinin and DPP-IV inhibitor were analyzed for total amide GLP-1 (SPE-MSD), and PYY (SPE-Luminex) according to manufacturers protocols. Significance P values for food intake, triglycerides, amide GLP-1 and PYY were calculated using a student's unpaired T-test.

### Lipomics

Jejunum tissue samples were sent to Tethys Bioscience for quantitation of lipid metabolites with a proprietary TrueMass Lipomic Panel. Significance P values were calculated using a student's unpaired T-test.

### Microarray Gene Expression

Tissue samples were submitted to Genelogic for RNA purification, probe synthesis and Affymetrix Genechip profiling. Affymetrix Rat Expression Array 230 2.0 chip was utilized to assess gene expression. For quality control, RNA degradation plots were generated for each CEL file. To assess potential RNA degradation, 3′/5′ ratios and their associated confidence intervals were evaluated [12]. Two techniques were used to distill the probe results into a small number of representative variables; Multidimensional scaling (MDS) [13] and Principal component analysis (PCA). These two techniques were applied to the data before and after Robust Mult-Array Average (RMA) [13] signal processing. During this processing, only the perfect match (PM) probe data were used; the mismatch (MM) probes were not used.

To assess differential expression of genes between groups of interest, a common statistical model was applied independently to each probeset. Gene expression for all sample types was analyzed on the log2 scale. Linear models were used to calculate t-statistics, which were subsequently adjusted using the moderated t-statistic procedure [14]. The Benjamini and Hochberg adjustment procedure [15] based on controlling the False Discovery Rate (FDR) was used.

### TaqMan Quantitative PCR

A subset of the observed gene changes on the Affymetrix array was confirmed by quantitative PCR analysis (Table S1). First strand cDNA was synthesized using the Applied Biosystems (ABI) High Capacity Reverse Transcription Kit according to the manufacturer's protocol. Gene expression was measured using a customized ABI 384-well TaqMan Low Density Array (TLDA) card and the TaqMan Universal PCR Master Mix (Applied Biosystems) on an ABI 7900HT sequence detector. Expression of target genes were normalized with the house-keeping genes 18S and validated with ACTB. Expression level of each gene was calculated by RQ method and expressed as “relative expression per 10^6^ 18S”.

### Gene Set Enrichment Analysis (GSEA)

Enrichment analyses were conducted using Gene Ontology (GO) groupings using a two-sided Fisher's Exact Test (i.e. hypergeometric test). The two-sided test can differentiate between enrichment and depletion of statistically significant results. For each test, the odds ratio (similar to a fold-change) is calculated using the observed and expected number of significant genes, as well as raw p-values. This technique accounts for the size of the groupings as well as the overlap between related groups in the hierarchy via conditioning. Each GO terms was scored using desirability functions [16] where the most desirable GO term would have large (absolute) fold-change, small p-value and a target group size of 20 genes.

### Causal Reasoning Engine Algorithm

The Causal Reasoning Engine (CRE) follows the general data model introduced in Pollard et al [9]. We utilized the CRE algorithm of Chindelevitch et al [10] which provides novel statistical measures to assess relevance of uncovered upstream regulators to plausibly interpret the observed expression changes. Briefly, the approach relies on a large collection of curated causal statements of the form:
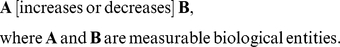



The biological entities can be of different types (e.g. phosphorylated proteins, transcript levels, biological process and compound exposure) and each statement is tied to accessible, peer-reviewed articles. For this work, we licensed approximately 450,000 causal statements from commercial sources (Ingenuity Systems and Selventa).

Each biological entity in the network and its assumed mode of regulation is a potential *hypothesis (e.g. predicted decrease in PPARG transcription activity)*. For each hypothesis, we can now compare all possible downstream transcriptional changes in the knowledge base with the observed transcriptional changes in the experiment. We consider two metrics to quantify the significance of a hypothesis with respect to our experimental data set, namely enrichment and correctness. The *Enrichment* p-value for a hypothesis h quantifies the statistical significance of finding *(#incorrect + #correct)* transcripts within the set of all transcripts downstream of h. The exact p-value can be computed by a Fisher's exact test. This is a standard approach in gene set enrichment methods and does not take the direction of regulation into account [17].

The *Correctness* p-value is a measure of significance for the score of a hypothesis h defined as (*#correct - #incorrect*). As desired, this score is high, if the number of correct prediction exceeds the number of incorrect predictions. To ensure statistical significance under a null model of randomly re-assigning up- and downregulated transcripts to arbitrary nodes, we compute the distributions for this score and derive appropriate p-values. Surprisingly, the distributions can be computed analytically in polynomial time using combinatorial programming approaches [10]. The Causal Reasoning Engine is implemented in the statistical programming language R [18] and uses the igraph package for representation of the network of causal assertions.

### Network Modeling of the CRE Hypotheses

**Figure 1:**
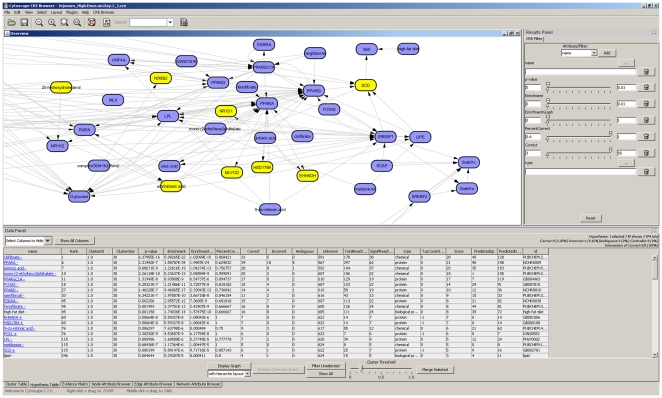
A screen shot of the cytoscape based causal reasoning engine browser showing the network overview window, hypothesis table and parameters filter panel.

The analysis results are communicated using the Causal Reasoning Browser (Figure 1), a Java application based on the open-source biological network viewer Cytoscape [19]. We have developed a plugin that enables browsing, clustering, merging, sorting and filtering of upstream hypotheses in conjunction with the relevant causal networks.

In the CRE browser an overview graph allows users to visualize hypotheses and examine their network relationships in the context of the causal relationships obtained from the literature based knowledgebase. Each edge in the graph is linked to an evidence statement directly obtained from the original peer reviewed article. Furthermore, each hypothesis can be expanded into a network showing all the causal relationships that qualified the hypothesis based on the gene expression changes from the experiment.

To facilitate the construction of biological networks from the generated hypotheses several analytical tools were developed. Alternative to manual selection of potentially related hypotheses a clustering tool uses cosine similarity metric and an average linkage method to group related hypotheses together. The average similarity for clustering can be adjusted by setting a cluster threshold to reflect the desired extent of similarity. Where 1 indicates hypotheses with identical supporting evidence and 0 indicates dissimilar hypotheses [20].

Once a subset of hypotheses is selected, either manually or using the clustering tool, similarity statistics are displayed in the browser. This includes the percent of unique gene expression changes correctly explained by this subset of hypotheses, overlap and contradictions. For each selected set of hypotheses a dynamic heatmap, the *evidence matrix*, is automatically generated whereby each cell in the heatmap represents the relationship between each hypothesis and each gene in this subset, if one exists. Finally, the browser enables the user to merge the hypotheses for further investigation of the underlying context, evidence statement and source literature.

## Results

### Food Intake and Plasma triglycerides level

**Figure 2:**
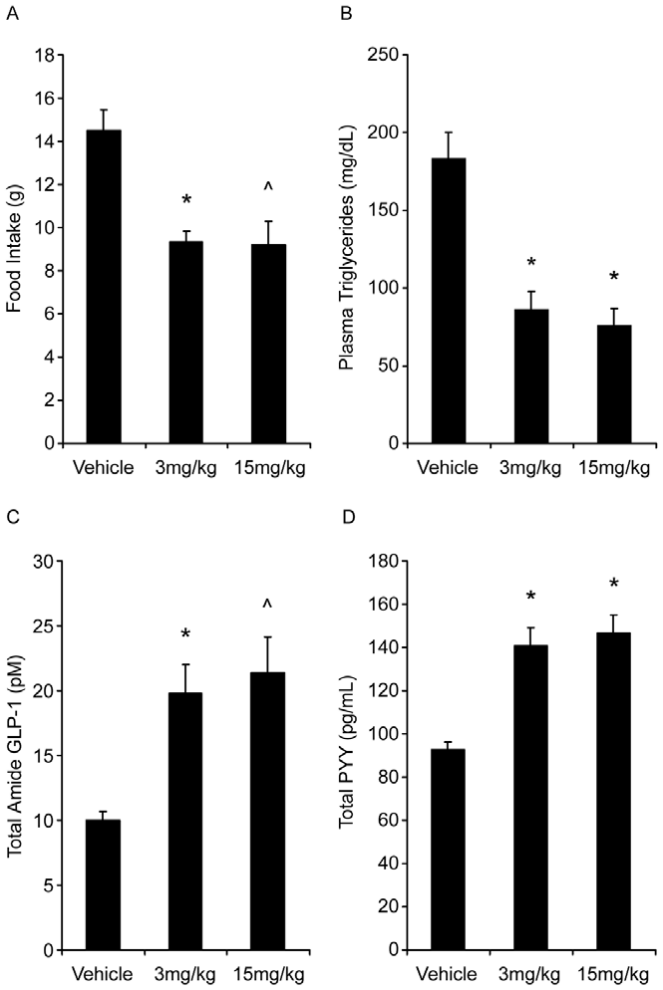
Food intake (A), plasma triglycerides (B), total amide GLP-1 (C) and total PYY (D) in response to a single dose of PF-04620110. Symbols indicate statistical significance from vehicle with *p-*value thresholds of <0.001 (*), <0.005 (∧), for n = 8 rats per treatment group.

In high fat habituated Sprague Dawley rats, mean six hour food intake was significantly decreased following administration of PF-04620110 by 40 and 37% at 3 and 15 mg/kg respectively relative to mean food intake of vehicle controls (Figure 2A). Six hour fed plasma triglyceride levels were significantly decreased by 53 and 59% at 3 and 15 mg/kg respectively compared to fed plasma triglyceride of vehicle controls following a single dose of PF-04620110 (Figure 2B). Total amide GLP-1 and PYY were also significantly increased in both dose groups (Figure 2C and 2D) at six hours.

### Gene expression analysis

A single dose of PF-04620110 resulted in 403 and 831 gene expression changes in the jejunum at 3 and 15 mg/kg, respectively, using an adjusted p-value <0.05 and fold change ≥1.3. Table S1 confirms a subset of the observed gene changes on the affymetrix array using quantitative PCR analysis. In contrast, no significant gene expression changes in the liver with the exception of one gene at high dose (data not shown). These data are consistent with the jejunum having the highest expression of DGAT1 in the Sprague-Dawley rat as evidenced by the ∼10-15 fold lower basal level of DGAT1 expression in the liver as compared to the jejunum (Data not shown).

### Gene Set Enrichment Analysis

Tables 1 and 2 show the top 10 most significant GO terms for the gene expression changes in the jejunum at high and low dose, respectively. The majority of the top ranking GO terms in the high dose group indicate several metabolic biological processes involving lipid, fatty acids, glycerol and cholesterol. However, in the low dose group there was minor indication of metabolic lipid changes.

**Table 1:** GO enrichment analyses for the 15 mg/kg group.

ID	Term	Raw p-Value	Odds Ratio	Size
GO:0006637 (BP)	acyl-CoA metabolic process	< 0.00001	12.838	14
GO:0005777 (CC)	peroxisome	< 0.00001	4.64	72
GO:0006071 (BP)	glycerol metabolic process	< 0.00001	16.615	10
GO:0006066 (BP)	alcohol metabolic process	< 0.00001	3.291	110
GO:0008203 (BP)	cholesterol metabolic process	0.0001	4.155	41
GO:0006869 (BP)	lipid transport	0.0001	3.404	59
GO:0006631 (BP)	fatty acid metabolic process	< 0.00001	2.478	126
GO:0019752 (BP)	carboxylic acid metabolic process	< 0.00001	1.79	455
GO:0045930 (BP)	negative regulation of progression through mitotic cell cycle	0.0002	35.548	6
GO:0004430 (MF)	1-phosphatidylinositol 4-kinase activity	0.0002	Inf	4

*The results are sorted by a scoring function that uses the p-value, odds-ratio and the size of the GO terms.

**Table 2:** GO enrichment analyses for the 3 mg/kg group.

ID	Term	Raw p-Value	Odds Ratio	Size
GO:0008191 (MF)	metalloendopeptidase inhibitor activity	0.0001	54.027	5
GO:0005604 (CC)	basement membrane	0.0001	6.368	28
GO:0006629 (BP)	lipid metabolic process	0	1.894	542
GO:0006457 (BP)	protein folding	0.0002	0.085	153
GO:0006066 (BP)	alcohol metabolic process	0.0002	2.074	257
GO:0005783 (CC)	endoplasmic reticulum	0.0006	1.718	466
GO:0016705 (MF)	oxidoreductase activity, acting on paired donors, with incorporation or reduction of molecular oxygen	0.0006	2.713	103
GO:0005885 (CC)	Arp2/3 protein complex	0.0007	17.7	7
GO:0005615 (CC)	extracellular space	0.0004	1.439	1419
GO:0005730 (CC)	nucleolus	0.001	0	94

*The results are sorted by a scoring function that uses the p-value, odds-ratio and the size of the GO terms.

### Causal Reasoning Engine Inferences and Network Modeling

Approximately 76% of the gene changes from each dose group mapped to biological entities in our knowledge base. Using the filtering functionality we applied the following cutoffs for the CRE generated hypotheses; correctness p-value <0.01, enrichment p-value <0.01, minimum number of correctly explained gene expression changes ≥3, percent correctly explained gene expression changes ≥60%. This resulted in a total of 79 and 160 hypotheses for the 3 and 15 mg/kg dose groups, respectively.

The CRE platform is developed with a set of analysis tools that enable flexible analysis schemes for evaluation of the biological significance of the hypotheses and integrating them into biological network models. However, for the purpose of this article we report the results from an example systematic approach utilizing some of the analysis tools we developed in the CRE browser. The basic premise for this approach is to preserve the context of the supporting evidence and employ it as the main driver for integrating the hypotheses into biological networks/models. To achieve this we started by grouping the hypotheses using the hypothesis clustering tool. We used a cluster threshold of 0.15 at which we see clustering of hypotheses that are not mere surrogates of one another. See tables 3 and 4 for examples of the top ranking largest clusters at cluster threshold 0.3 and 0.15, respectively. Table 3 shows the top two clusters from the 15 mg/kg group at cluster threshold 0.3 to be primarily composed of redundant hypothesis such as decrease in *PPARA-* and number of its ligands, and decrease in a number of SREBF family members. However, in table 4 the largest and highest ranking cluster for the 15 mg/kg group at cluster threshold 0.15 is indicative of decreased PPAR signaling, decreased lipids (*high-fat diet-, diacylglycerol-, lipid-*) increased fatty acid oxidation enzymes (*EHHADH+, HSD17B4+*). The second ranking cluster comprises hypotheses indicative of decreased sterol regulators (*SREBFs-*, *SCAP-*) and cholesterol trafficking (*NPC1-*), and decreased insulin resistance (*hyperinsulinism-,* supported by causal relations from studies of high fat diet in insulin resistance animal model [21]) and decreased glucose response regulators and glucose dependant activators of carbohydrate response element (*MLX-, MLXIPL-, Carbohydrate-*).

**Table 3:** Top two clusters for the high dose at cluster threshold 0.3.

Hypothesis	Hypothesis Rank	Cluster ID	Cluster Size	Correctness p-value	Enrichment p-value	Percent Correct
*Clofibrate-*	1	1	5	6.38E-16	5.99E-22	0.87
*PPARA-*	6	1	5	2.32E-07	1.59E-34	0.63
*Pirinixic acid-*	7	1	5	9.09E-09	1.23E-19	0.76
*Gemfibrozil-*	33	1	5	6.34E-07	1.98E-10	0.85
*Fenofibrate-*	45	1	5	0.001993	3.38E-13	0.67
*SREBF1-*	12	12	5	1.28E-08	1.16E-12	0.86
*Srebf1a-*	19	12	5	2.55E-07	2.65E-10	0.88
*Srebf1c-*	60	12	5	0.0002758	2.32E-05	0.89
*SREBF2-*	76	12	5	0.0036389	3.36E-07	0.75
*SCAP-*	76	12	5	0.0013972	1.73E-06	0.8

The sign following the hypothesis name indicates the CRE predicted directionality (*+*  =  predicted increase and *-*  =  predicted decrease).

**Table 4:** Top two clusters for the high dose at cluster threshold 0.15 after excluding redundancies.

Hypothesis	Hypothesis Rank	Cluster ID	Cluster Size	Correctness p-value	Enrichment p-value	Percent Correct
*PPARA-*	6	1	30	2.32E-07	1.59E-34	0.63
*MEHP- (PPARG modulator)*	10	1	30	2.26E-10	5.24E-13	0.91
*PPARGC1A-*	11	1	30	1.32E-08	8.94E-09	0.89
*FOXA2-*	15	1	30	4.25E-07	1.21E-11	0.82
*PPARD-*	27	1	30	1.46E-07	9.46E-17	0.74
*ESRRA-*	45	1	30	0.0022058	2.06E-12	0.68
*high-fat diet-*	45	1	30	0.0011581	1.74E-14	0.67
*EHHADH+*	60	1	30	3.51E-08	5.59E-08	1
*HSD17B4+*	60	1	30	3.51E-08	5.59E-08	1
*9-cis-retinoic acid-*	76	1	30	0.0062967	7.64E-06	0.75
*PCK1+*	76	1	30	3.30E-09	4.54E-09	1
*LPL-*	115	1	30	0.0009964	1.61E-06	0.78
*meldonium-*	115	1	30	8.68E-07	1.17E-06	1
*SCD+*	115	1	30	0.0001995	5.89E-06	0.86
*quercetin-*	164	1	30	0.0006924	0.0025397	1
*TFAM-*	164	1	30	0.0006092	0.0013388	1
*Prasterone-*	164	1	30	0.0003124	0.0004421	1
*diacylglycerol-*	246	1	30	0.0003967	0.0004851	1
*lipid-*	246	1	30	0.0046444	5.29E-05	0.8
*SREBF1-*	12	12	15	1.28E-08	1.16E-12	0.86
*RXRA-*	19	12	15	8.69E-08	3.42E-13	0.79
*MLX-*	19	12	15	3.86E-10	1.10E-09	1
*25-hydroxycholesterol+*	76	12	15	2.35E-05	1.05E-06	0.88
*SREBF2-*	76	12	15	0.0036389	3.36E-07	0.75
*SCAP-*	76	12	15	0.0013972	1.73E-06	0.8
*hyperinsulinism-*	164	12	15	0.0001294	0.0003134	1
*essential fatty acid-deficient diet-*	164	12	15	1.94E-05	2.50E-05	1
*NPC1-*	246	12	15	0.0003757	0.0019058	1
*MLXIPL-*	246	12	15	0.0006679	0.0008302	1
*cafeteria diet-*	246	12	15	0.0012353	0.0026628	1
*carbohydrate-*	246	12	15	0.0059513	0.0090411	1
*THRSP-*	246	12	15	0.0003967	0.0004851	1

The sign following the hypothesis name indicates the CRE predicted directionality (*+*  =  predicted increase and *-*  =  predicted decrease).

**Figure 3:**
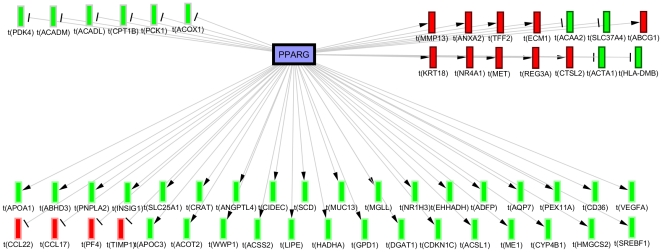
Causal network shows the experimental gene expression changes enriched for the *PPARG-* hypothesis in the 15 mg/kg group. 36 genes are consistent with the predicted decreased directionality (bottom), 14 are contradictory (top right) and 6 are ambiguous due to contradictory literature (top left). (Blue nodes  =  predicted decrease, Red nodes  =  observed mRNA decrease, Green nodes  =  observed mRNA increase).

In order to understand the context of the hypotheses we investigate the nature of the causal relationships supporting them by referring to their original studies. This is especially helpful for molecular hypotheses with a broad range of context dependant biological functions such as *PPARG-* (Rank = 5, Correctness p-value = 1.94E-7, Enrichment p-value = 1.21E-26). For example, investigating the causal relationships from the *PPARG-* subnetwork (Figure 3) reveals that ∼50% of the supporting assertions consistent with the predicted directionality were derived from studies on induction of adipogenesis [22], [23], the majority of which in context of adipogenic steatosis due to PPARG overexpression [24]. On the other hand, many of the assertions inconsistent with the predicted directionality originated from studies of PPARG insulin sensitization in Zucker diabetic rat model [25] and cholesterol efflux in macrophages [26].

**Figure 4:**
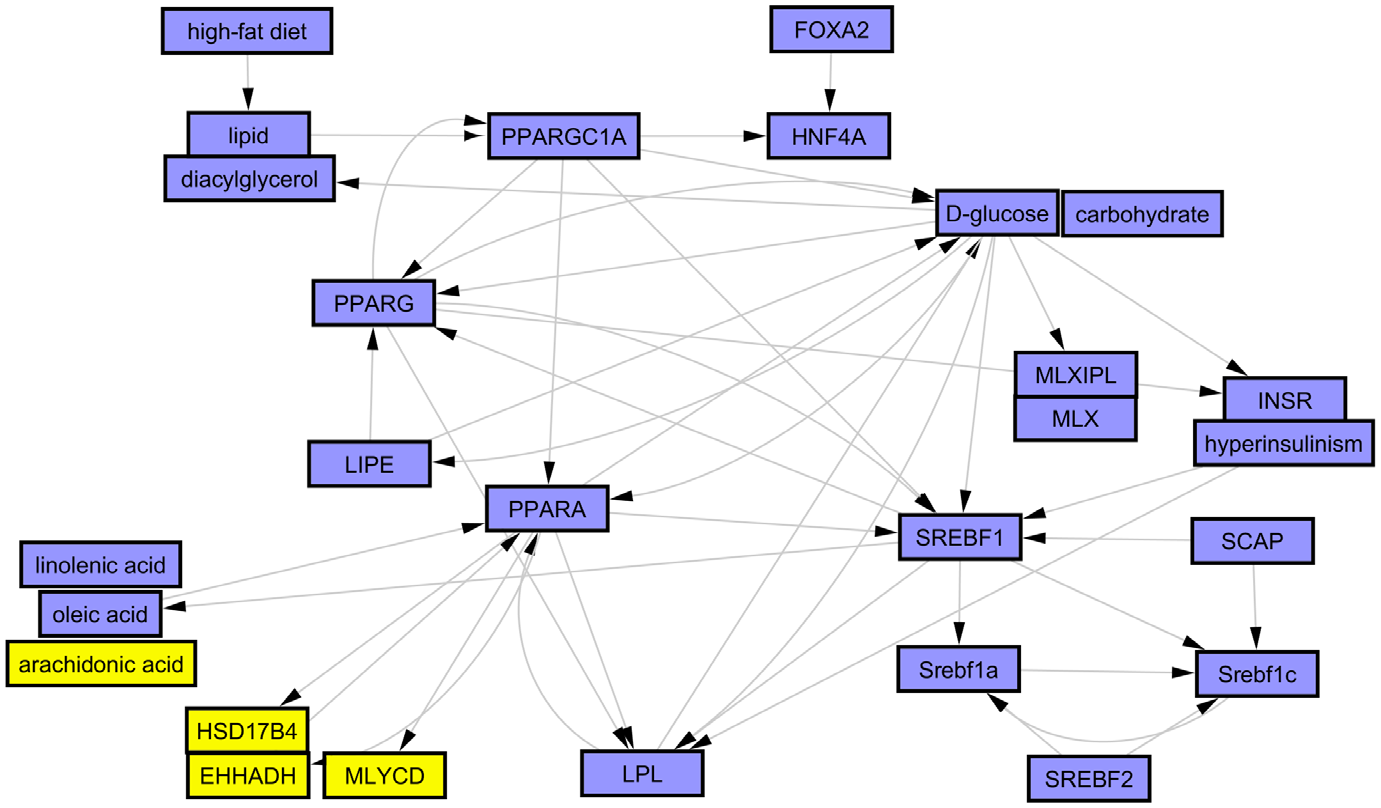
Summary overview graph showing the biological model constructed for reversal of high fat diet, decreased insulin resistance and changes in fatty acid metabolism in response to PF-04620110 treatment. (Blue nodes  =  predicted decrease, Yellow nodes  =  predicted increase).

**Figure 5:**
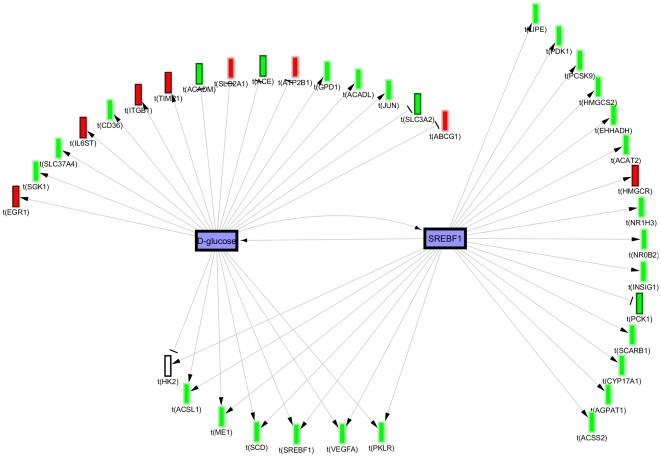
Merged causal graph showing potential positive reinforcing interactions between *glucose-* and *SREBF1-* supported by evidence from studies on its role in mediating hyperlipidemia in response to high fat diet [**27**]. (Blue nodes  =  predicted decrease, Red nodes  =  observed mRNA decrease, Green nodes  =  observed mRNA increase).

**Figure 6:**
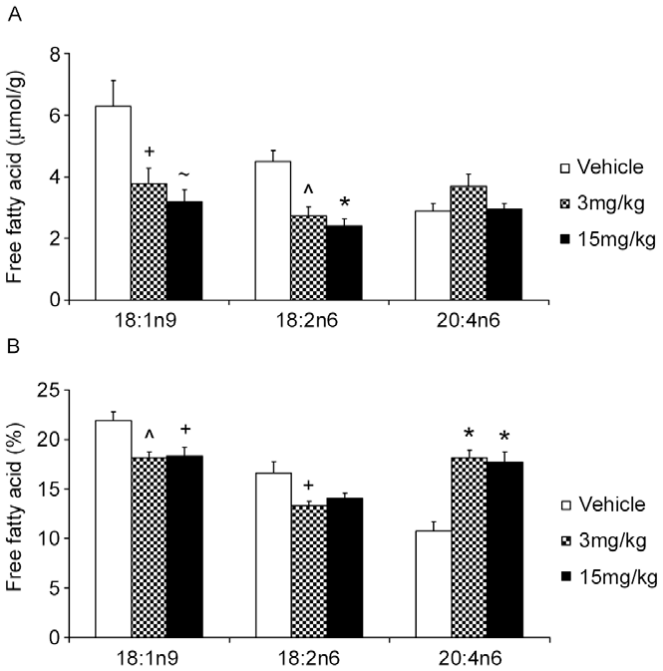
Results from lipomics analysis showing effect of PF-04620110 on tissue levels of two of the most abundant free fatty acids: Oleic 18:1n9 and Arachidonic acid 20:4n6 in rat jejunum. Represented in raw values (umol/g tissue, panel A) and normalized values (% of total free fatty acids, panel B). Symbols indicate significance from vehicle with P value thresholds of <0.001 (*), <0.005 (∧), <0.01 (∼), <0.05 (+) for n  =  8 rats per treatment group.

Lastly, we constructed biological networks primarily guided by hypothesis clustering and investigation of the underlying evidence and the potential inter-hypothesis causal relations from the causal graph overview. These biological models (summarized in Figure 4) support 3 major effects of PF-04620110; reversal of the high fat diet and decreased hyperlipidemia, decreased insulin resistance and decreased glucose, and altered fatty acid metabolism. The key high-fat diet responsive regulators supported by the causal evidence are *PPARG-* (see above) and *SREBFs-* (e.g. some of the assertions supporting *SREBF1-* are obtained from a study demonstrating its role in mediating the hyperlipidemic response to high fat diet [27]). Glucose metabolism is represented by a network of hypotheses indicative of decreased glucose levels, decreased glucose response activators and decreased insulin resistance. The glucose metabolism network appears to be secondary to decreased lipids; however, there exists causal interactions with several lipid network components positively reinforcing both networks as evidenced by edges from the overview graph and investigating the context of overlapping assertions using the merge hypotheses function (Figure 5). The third network indicates decrease in some fatty acids like linolenic and oleic acid but increase in arachidonic acid. Lipomics analysis of corresponding jejunum tissue from the same rats confirmed the predicted changes in these free fatty acids. Figure 6 shows the depletion of oleic acid C18:1n9 and the enrichment of arachidonic acid C20:4n6 in the jejunum with DGAT1 inhibition. The less abundant linolenic acid was also significantly depleted -2.4 fold (umol/g tissue) for both dose groups. Finally, there is also support for a number of nuclear receptors and co-regulators to cooperate in more than one of the above 3 main effects (*HNF4A-, FOXA2- and PPARGC1A-*).

## Discussion

The objective of this study was to employ a novel computational platform to gain mechanistic insight into the molecular changes induced by pharmacological inhibition of DGAT1. Acute gene expression changes were utilized to infer multiple overlapping molecular regulators of lipid and carbohydrate metabolism predictive of benefits of DGAT1 inhibition such as lipid lowering and improved insulin sensitivity. Our analysis allows us to postulate the molecular network conferring these metabolic benefits to better understand the mechanism of action for pharmacological inhibition of DGAT1.

Our understanding of the physiologic role of DGAT1 stems largely from studies of genetically modified mice that lack DGAT1 from birth. It is noteworthy that this analysis focused on transcriptomics in the jejunum elicited by the administration of a pharmacological inhibitor of DGAT1 in an adult rat which suggests similar molecular phenotype to DGAT1 knockout mice. Recently, DGAT1 knockout mice were shown to have decreased expression of PPARalpha, gamma and delta as well as target genes suggestive of reduced lipid uptake and metabolism and increase glucose uptake [28] which is consistent with our top ranking hypotheses. Additionally, DGAT-1 deficient mice demonstrate resistance to weight gain on high fat diet, improved insulin sensitivity and a lower percentage of oleic acid in their skeletal muscle and adipose tissue triglyceride [29]. Again, our CRE generated hypotheses identified reversal of high fat diet, reduced insulin resistance and decreased oleic acid. These data support the notion that the intestine is an important tissue involved in whole body insulin sensitivity diet-induced obesity. Insulin resistance in the intestine has been associated with increased apolipoproteins, chylomicrons, de novo lipogenesis, and increased fatty acid and cholesterol uptake via CD36 and SCARB1 [30]. In our study not only was triglyceride synthesis decreased via inhibition of the target, but transcription of the key apolipoproteins for chylomicron synthesis (ApoB, ApoA I, ApoA IV, and ApoC III) were reduced. Of these Apo CIII was the most dramatic (see Table S1) with greater that a 5 fold reduced expression at the high dose. The expression and secretion of ApoC III is increased in insulin resistant states and plasma circulating levels are higher in metabolic syndrome and type II diabetes [31]. Finally, Lee et al demonstrated that intestine specific expression of DGAT1 in the DGAT1 deficient mice prevented the knockout mouse from being resistant to diet induced obesity [6].

In contrast, DGAT1 knockout mice are hyperphagic [29], [32]; whereas, administration of PF-04620110 results in a decrease in food intake. Our working hypothesis is that elevated levels of incretin hormones glucagon-like peptide-1 (GLP-1) and peptide YY (PYY) are at least in part mediating this response (Figure 2). It is our belief that decreased food intake is an integral part of the mechanism of action driving a metabolically favorable profile following pharmacological inhibition of DGAT1 and thereby did not try to dissociate food intake dependent effects from food intake independent effect in our analysis. Normal lipid absorption entails the breakdown of dietary triglyceride into free fatty acids and 2-monoacylglycerol by pancreatic lipases in the lumen of the small intestine. This allows transport of the free fatty acids into the enterocytes where they can be re-esterified and packaged into chylomicrons for delivery to the circulation. Clearly the major role of DGAT1 in triglyceride synthesis and intestinal lipid absorption has been demonstrated [33] with DGAT1 accounting for 89% of triglyceride synthesis in rat intestinal membranes. Theoretically, DGAT1 inhibition would cause an immediate build up of its substrates, diacylglycerol and free fatty acids. Polyunsaturated fatty acids have been demonstrated to decrease the expression of lipogenic genes via SREBP promoter elements (SRE) [34]. Therefore DGAT1 inhibition would result in decreased lipogenesis in the intestine driven by an excess of free fatty acids. There has been mounting evidence in high fat diet rodent models and humans supporting a negative impact of de novo lipogenesis and monounsaturated fatty acid synthesis on insulin sensitivity [21], [35], [36]. Mice fed high fat western diet for one week demonstrate a robust increase in the expression of intestinal SREBF1 and SCD-1, and develop insulin resistance with little change in hepatic gene expression [21], [35], [36]. Coincidentally, SREBF1 and SCD1 where robustly down regulated in the jejunum but unchanged in the liver with DGAT1 inhibition. Furthermore CRE hypotheses for reduced SREBF1, PPARa, RXR, MLX, and PGC1a all suggest a decrease in fatty acid synthesis, while the decrease in SCD1 may be contributing to the depletion of oleic acid, and secondary enrichment in arachidonic acid (Figure 6). Recent evidence has indicated a benefit for a high ratio of C20-C22 PUFAS to saturated and monounsaturated fatty acids for improved glycemic control and insulin sensitivity [37]. Thus an additional effect of DGAT1 inhibition would be the insulin sensitizing effect of enriched very long chain PUFA.

The Causal Reasoning approach has the advantage of providing detailed molecular hypotheses on potential causal drivers of observed expression changes. Each assertion can be followed back to the primary literature providing confidence to the researcher to follow-up on the computational predictions. In some cases the predicted direction of the CRE hypothesis may conflict with the observed direction of the transcript change. For example, a CRE hypothesis of decreased *CFTR* protein and/or activity conflicts with the observed increase in transcripts for CFTR as well as Annexin 2 and S100A10 that complex with CFTR enabling its function [38]. The literature evidence supporting the *CFTR* hypothesis came from two studies in CFTR knockout mice [39], [40]. Regulated genes in this context may include compensatory and/or regulatory feedback gene expression changes which in turn may complicate the interpretation of some of the CRE hypotheses. One possibility is that a CRE hypothesis may represent protein level or activity which is not necessarily reflective of the mRNA level or that the CRE hypothesis is based on gene changes in response to an initial decrease in CFTR protein or activity that led to feedback increase in transcript level hence reflecting an earlier temporal event. Another example is the CRE hypotheses for increased fatty acid oxidation enzymes Enoyl-CoA hydratase (EHHADH), and hydroxysteroid (17-beta) dehydrogenase 4 (HSD17B4). Both of these hypotheses are supported by the same transcript evidence from a single literature source [41]. Moreover, the same transcript evidence is completely subsumed under the much higher ranking hypothesis of decreased PPAR alpha, which includes decreased transcription of EHHADH and HSD17B4 which could be an effect of a feedback loop.

Clearly, the hypotheses as well as the resulting model can only be as good as the underlying causal relationships. Consequently, the method is unlikely to uncover completely novel areas of biology. However, it can provide novel insights by reporting upstream drivers to be relevant in a certain context. As efforts to curate larger parts of the biomedical literature are underway, we expect the power of the approach to increase.

We have employed the causal reasoning approach as a means of visualizing an extensive and diverse set of gene expression changes to generate high level molecular hypotheses that will enable a better understanding of the anti-adipogenic and anti-diabetic benefits derived following pharmacological inhibition of DGAT1. Additionally, this analysis has allowed us to understand the advantages and limitations of causal reasoning. The approach has allowed us to confirm in a systematic fashion that pharmacological inhibition of DGAT1 in adult rats generates molecular hypotheses that are consistent with the metabolically beneficial phenotype of mice lacking DGAT1.

## Supporting Information

Table S1 Fold changes as measurement by Affymetrix microarray and by TaqMan QPCR (NA  =  data not available).
(XLS)
